# Distance to Thrombus in acute middle cerebral artery stroke predicts basal ganglia infarction after mechanical thrombectomy

**DOI:** 10.18632/oncotarget.13280

**Published:** 2016-11-10

**Authors:** Benjamin Friedrich, Donald Lobsien, Christian Maegerlein, Silke Wunderlich, Claus Zimmer, Johannes Kaesmacher, Justus Kleine

**Affiliations:** ^1^ Department of diagnostic and interventional Neuroradiology, Klinikum rechts der Isar, Munich, Germany; ^2^ Department of Neuroradiology, University Hospital Leipzig, Leipzig, Germany; ^3^ Department of Neurology, Klinikum rechts der Isar, Munich, Germany

**Keywords:** distance to thrombus, stroke, basal ganglia, mechanical thrombectomy

## Abstract

**Background and Purpose:**

This study examines if involvement of the lenticulostriate arteries (LSAs) in MCA stroke and consecutive infarction of the basal ganglia can be predicted by the exact occlusion site, as determined in pre-interventional CT or MRI imaging.

**Methods:**

Retrospective analysis of 212 patients with acute isolated MCA occlusions treated with mechanical thrombectomy. The occlusion site was assessed using the Distance to Thrombus (DT). Affection of LSAs by the occlusion was determined by analysis of pre- and post-interventional DSA runs. Infarction of the striatocapsular region was evaluated in post-interventional imaging.

**Results:**

DT showed a highly significant correlation with the affected LSA group (*ρ* = 0.747; *P* < 0.001). In a ROC analysis, DT could predict affection of the LSAs with an area under the curve (AUC) of 0.903. Additionally, DT could predict an infarction of the striatocapsular region with an AUC of 0.824. In a stepwise regression analysis for striatocapsular infarction including DT, age, time from symptom onset to recanalization and recanalization success, only DT proved to be an independent predictor.

**Conclusion:**

In MCA stroke, the exact site of the occlusion as measured by DT independently predicts the involvement of LSAs and subsequent striatocapsular infarction with high sensitivity and specificity.

## INTRODUCTION

Mechanical thrombectomy (MT) has been shown by five prospective randomized trials [1–5] to be superior to i.v. thrombolysis alone, and constitutes a paradigm shift in the treatment of acute anterior circulation stroke caused by large vessel occlusion (LVO). However, the capacity of MT to salvage ischemic tissue in acute LVO stroke has limitations. Specifically, in middle cerebral artery (MCA) occlusion, infarction of dependent tissue in the striatum will almost always occur - even after fast and successful recanalization - if the occluding thrombus extends across the orifices of the lenticulostriate arteries [6]. These arteries, which mainly arise, in an orderly manner, from the MCA M1 segment and, to a lesser extent, from the A1 segment of the anterior cerebral artery (ACA) [6, 7], are “endarteries” that do not anastomose with other vessels [8]. Hence, if they are blocked by thrombus in the MCA, blood flow to the dependent LSA-supplied subterritories will be shut off completely, virtually inevitably leading to subsequent infarction [6]. LSAs are routinely visualized on digital subtraction angiography (DSA), but generally not visible in standard acute stroke imaging modalities, i.e. Computed Tomography (CT) or Magnetic Resonance Imaging (MRI).

The aim of the present study was to analyze if involvement of the LSAs and consecutive infarction of the basal ganglia in acute MCA stroke can be predicted by the exact occlusion site, as determined by the “distance to thrombus” (DT) [9] in pre-interventional CT or MRI imaging.

## RESULTS

**Figure 1:**
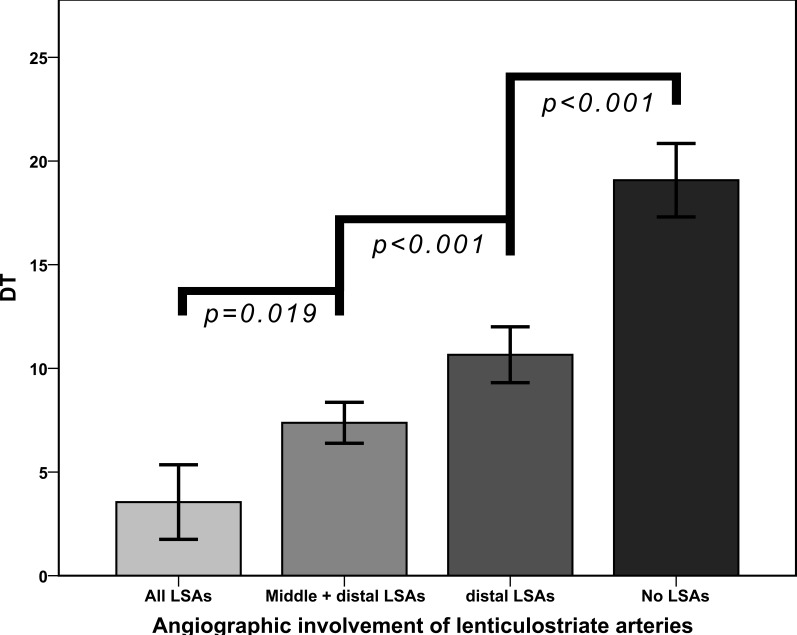
Involvement of different subgroups of lenticulostriate arteries (LSAs) depending on the exact occlusion site as expressed by DT DT can discriminate precisely the affected LSA subgroups. Data are given in mean +/- SD.

**Figure 2:**
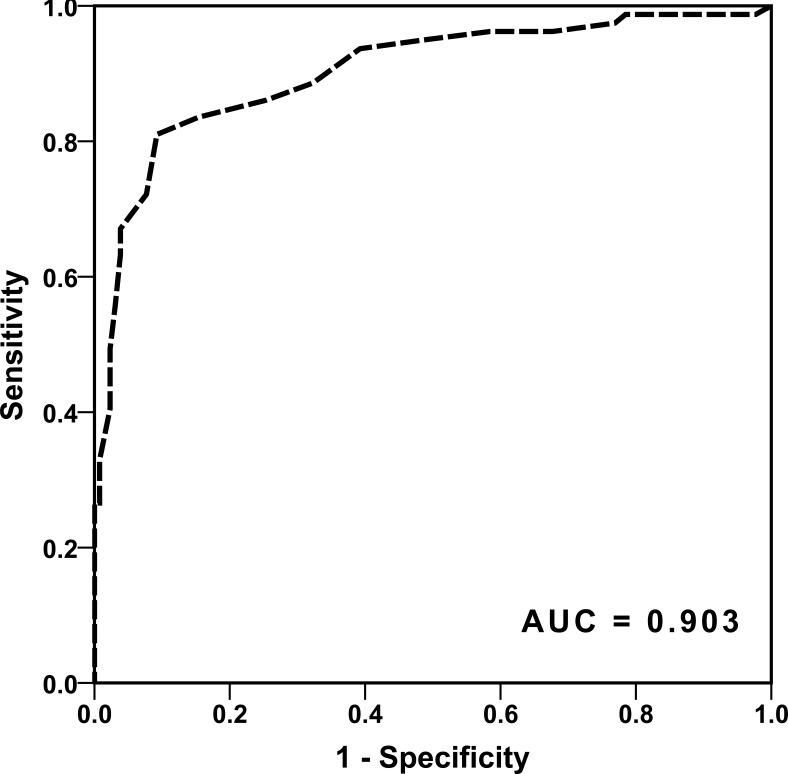
ROC analysis if DT can predict an involvement of the perforators in the setting of acute MCA occlusion Here DT can predict the involvement of the LSAs with an area under the curve of 0.903

**Figure 3:**
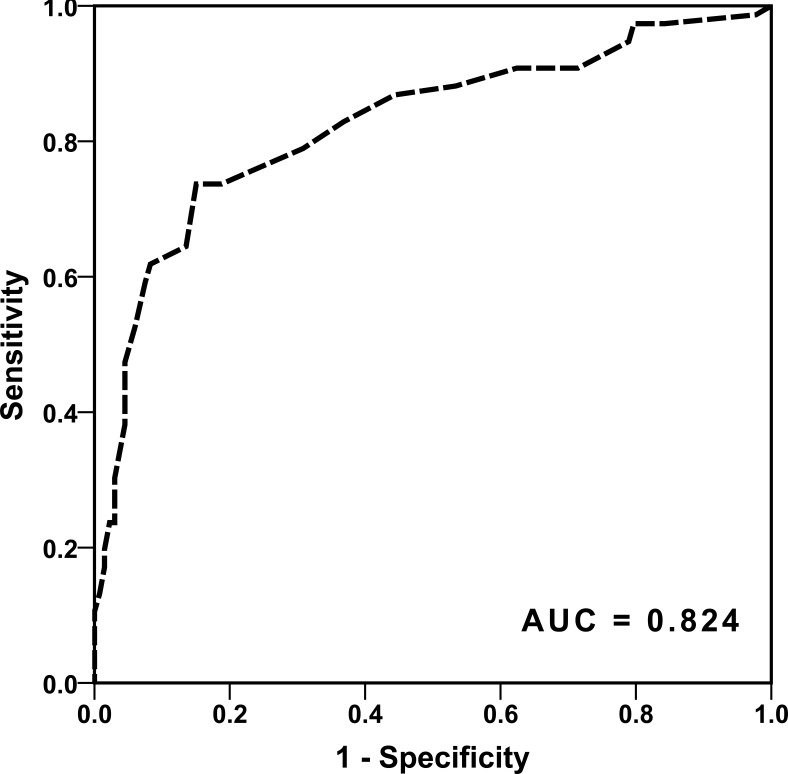
ROC analysis if DT can predict a post-interventional infarction of the striatocapsular region DT once more can predict an infarction of the striatocapsular region with an area under the curve of 0.824.

Applying the above-mentioned inclusion and exclusion criteria we were able to include 211 patients. Patients had an average age of 72 +/- 15 years and were predominantly female (54.8%). 94.3% of the patients were scanned with a pre-interventional CT-Angiography, 5.7% by MR-Angiography. The majority had a severe neurological deficit with a median National Institute of Health Stroke Scale (NIHSS) of 14 (IQR 11 -17). All patients showed an initial TICI score of 0, indicating a complete occlusion of the vessel. 82% of the patients could be recanalized successfully with a post-interventional TICI Score of 2b or 3 (35% TICI 3). No patient had a relevant intracranial stenosis of the M1 segment with the subsequent need for permanent emergency stenting. The average time from symptom onset to recanalization was 248 minutes +/- 73 minutes. On pre-interventional imaging, an average DT of 12 +/- 8 mm was determined. DT showed a highly significant correlation with the affected LSA groups (*p* = 0.747; *P* < 0.001). Performing an ANOVA on ranks with a Tukey posthoc test, the different LSA groups showed a significant different average DT. While the involvement of the proximal LSAs occurred at a DT of 3.5 +/- 0.9, the middle LSAs were affected at a DT of 7.4 +/- 0.5. The lateral LSAs showed an involvement if the DT was 10.6 +/- 0.7. No involvement of LSAs could be detected with a DT of 19 +/- 0.9 (Figure 1). Performing a ROC analysis for the general affection of the LSAs - expressed by DT - we could find an area under the curve (AUC) of 0.903 (Figure 2). In the analysis of the corresponding Youden Index, a DT of 15mm showed the highest sensitivity and specificity with 96.2% and 71% respectively. When we analyzed the ability of DT to predict an infarction of the striatocapsular region by a ROC analysis we calculated an AUC of 0.824 (Figure 3). The corresponding Youden Index showed the highest discrimination for SCI infarction at a DT of 13mm with a sensitivity of 85% and a specificity of 74%. Performing a stepwise regression analysis for SCI infarction including DT, age, time from symptom onset to recanalization and recanalization success, only DT proved to be an independent predictor (*P* < 0.001). Despite the fact that DT could significantly discriminate between the LSA groups affected by the occlusion, DT failed in differentiating the subtypes of post-interventional SCI infarction (SCI-1 *vs*. SCI-2 *P* = 0.819 / SCI-1 *vs*. SCI-3 *P* = 0.418).

**Table 1:** Patient characteristics

**Age (min./max.)**	72 +/- 15 years (20/97)
**Sex**	54.8% female
**Imaging modality**	94.3% CT-Angiography
**Mean NIHSS (IQR)**	14 (11 – 17)
**Symptom onset to recanalization (min./max.)**	248 +/- 73 minutes (120/537)
**Successful recanalization (TICI 2b/3)**	82%
**‘bridging’ i.v. thrombolysis**	63%
**Distance to thrombus (min./max.)**	12 +/- 8 (1/38)

## DISCUSSION

In the present study, we were able to show that the exact location of the vascular occlusion in acute MCA stroke measured by DT can predict both the involvement of the lenticulostriate perforators as well as infarction within the striatocapsular region with high sensitivity and specificity.

The standard in imaging for suspected acute ischemic stroke due to a large vessel occlusion is either by computed tomography in combination with CT-Angiography or by MRI in combination with MRA. Both imaging modalities can reliably detect occlusions of the large intracranial vessels. Anyhow, both modalities are strongly limited in visualizing small and smallest vessels like the LSAs [10]. It is known that proximal occlusions of the M1 segment are associated with a worse clinical outcome than more distal vessel occlusions [9, 11]. One explanation could be the involvement of the LSAs with subsequent striatocapsular infarction [6]. The use of DT for prediction of the involvement of the LSAs provides a new tool in the assessment of LSA affection in acute MCA stroke despite their non-visibility in standard acute stroke imaging. DT, however, could not just predict the involvement of the LSAs but also infarction within the striatocapsular region. As infarction of this region may be of substantial clinical relevance, assessment of the likelihood of such an imminent event may be of considerable value in therapeutic decisions. DT could predict the infarction of the striatocapsular region independently of patient characteristics such as age and even independently of therapeutic success parameters such as time to or rate of successful recanalization. This indicates that infarction of the basal ganglia occurs within a very short time window after vascular occlusion, which is in conjunction with previously published results [6, 12]. Our results corroborate that the great benefit of endovascular treatment by mechanical thrombectomy in proximal MCA occlusion is not based on the salvage of the basal ganglia, but on salvage of more distal regions [6]. Consequently, recognizable infarction of the basal ganglia in pre-interventional imaging should perhaps not be viewed as a strict contraindication against MT, since the main goal of MT cannot be the rescue of the striatocapsular territories, as suggested previously [6].

Despite the fact that DT could discriminate the involvement of different perforator subgroups, we were not able to predict the corresponding subgroup of infarction of the striatocapsular region. A related previous observation is that striatocapsular infarctions are almost never smaller, but not infrequently larger, often extending into more “proximal” LSA subterritories, than predicted by the exact LSA involvement [6]. The most likely explanation for this discrepancy is early distal migration of the occluding thrombus (prior to imaging) and a resulting discrepancy between depicted LSA involvement and infarct pattern. Further studies are needed to clarify this phenomenon.

Previous detailed analyses of occlusion sites, individual vascular anatomy, and striatocapsular infarction patterns in individual patients with MCA occlusion showed that individual anatomical variants in LSA supply schemes could account for most of the relatively rare cases, in which striatocapsular infarctions were actually smaller than predicted by a superficial “first-glance” assessment of occlusion sites [6]. Irrespective of the relevance of such anatomical vascular supply variants in some individuals, the present data suggest that the vascular supply of the striatocapsular region, overall, is quite homogenous across patients, as DT completely ignores such individual deviations from normal LSA anatomy. The relative homogeneity of the LSAs could be shown clearly by the distinction of affected groups regarding the exact occlusion site as expressed by DT (Figure 1). These data suggest that the charmingly simple measure of DT may allow reasonably accurate prediction of LSA involvement also in individual patients.

Our study has several limitations. First, it is a retrospective study. Anyhow, the analysis was performed based on a prospective database with all consecutive patients fulfilling the defined inclusion criteria minimizing the limitations of a retrospective analysis. Second, DT was analyzed by only one rater. In previous studies the measurement of DT could be shown to have an excellent inter-rater agreement if performed by multiple readers [9, 13], therefore it is valid to assume that a single experienced reader can analyze DT with high precision. Third, it is possible that the occlusion site, defined by DT, is not the real beginning of the clot but merely a combination of the vascular occlusion in combination with an area of limited opacification in CT-Angiography due to stasis in blood flow proximal to the thrombus. One argument against this assumption is that when we analyzed only those cases with pre-interventional MRI-based time of flight (TOF) angiography (*n* = 12), the results, regarding the correlation between DT and affection of the different perforator groups (*p* = 0.712; *P* < 0.001), were essentially similar in comparison to cases assessed by CT-Angiography. Additionally, in a recent study, it was shown that the beginning of the thrombus visualized by MRI-based Susceptibility-weighted imaging and the occlusion site in the TOF-Angiography do not differ signficantly [14], implying that there is no relevant area of stasis in blood flow proximal to the thrombus in acute MCA stroke. On the other hand, even if there was an area of stasis in blood flow proximal to the clot, this stasis in blood flow would most likely have the same effect on the vascular blood supply be the lenticulostriate perforators as a blocking thrombus itself.

## CONCLUSIONS

In acute MCA stroke, DT independently predicts the involvement of basal ganglia perforators and subsequent infarction of the striatocapsular region with high sensitivity and specificity. This may contribute to early estimation of the potential neurological deficit of the patient and help to guide treatment decisions.

## MATERIALS AND METHODS

### Study population

A retrospective analysis of a prospectively collected database including all consecutive patients (01/2007-06/2016) treated with mechanical thrombectomy (MT) for an isolated occlusion of the MCA were included (*n* = 403). We excluded patients with inadequate imaging to assess infarct extent (*n* = 72), patients without pre-interventional CT- or MR-Angiography (*n* = 113) and patients with insufficient image quality to analyze DT adequately (*n*= 7). This study is in accord with the 1964 declaration of Helsinki and its later amendments and was approved by the local ethics committee. Written informed consent of individual patients for this anonymized, retrospective observational study was waived according to institutional guidelines.

### Endovascular therapy

Patients with acute large vessel occlusion stroke were considered eligible for MT if there were no extensive early ischemic signs in pre-interventional imaging (ASPECTS > 5) in a time window of less than 6 hours from symptom onset. No age limit was applied. Intravenous thrombolysis with recombinant tissue plasminogen activator (IV rtPA) was applied as bridging therapy in all eligible patients.

MT was performed with standard techniques, as described previously [6]. Procedures involved stent retrievers and direct aspiration techniques with large lumen aspiration catheter (ADAPT) used as a standalone technique or in conjunction with stent retrievers.

Successful recanalization was defined as Thrombolysis in Cerebral Infarction (TICI) 2b or 3 on the TICI scale, with TICI 2b defined as reperfusion in more than two-thirds of the initially involved territory according to the original publication by Higashida et al [15].

### Distance to thrombus

DT was measured as previously described [9, 13]. In short, in pre-interventional obtained CT-Angiography or MR-Angiography scans - reformatted to a 10mm coronal plane MIP projection - the distance from the carotid-T to the thrombus was measured along the course of the MCA in a curved line by an attending in Neuroradiology with more than 6 years in acute stroke image interpretation. The result in mm was the DT.

### Involvement of LSAs and infarct pattern

The exact thrombus location was graded depending on which, if any, orifices of the LSA groups (proximal, middle, lateral) were occluded, as described previously [6]. In order to assess the LSA involvement a synopsis of pre- and post-interventional DSA runs was used. The respective extent of infarction in the striatocapsular region was evaluated on post-interventional imaging based on a classification system of striatocapsular subterritories suggested by Kleine et al. [6], which was shown to correspond well with the respective territory supplied by the different LSA subgroups. In short, a striatocapsular type 1 infarction (SCI-1) involves the complete caudate nucleus (including the dorsal parts of the caudate head) and the putamen and corresponds to an occlusion of all LSA subgroups. Striatocapsular type 2 infarctions (SCI-2) spare the rostral tip of the putamen and the caudate head and are caused by an occlusion of the middle and lateral LSA subgroups, while the proximal LSA subgroup is spared. A striatocapsular type 3 infarct (SCI-3) is confined to the dorsal part of the putamen and the dorsal tail of the caudate nucleus and correspondingly is accounted for by an occlusion of the orifices of the lateral perforator subgroup [6]. All of these infarct patterns involve the dorsal genu of the internal capsule at the level or shortly before the fibertracts pass through the posterior caudatolenticar bridges of gray matter [16].

### Statistical analyses

Statistical analyses were performed using SPSS software version 20 (IBM, Armonk, NY, USA). Differences between groups were analyzed by a univariate analysis using analysis of variance on ranks with a Tukey posthoc analysis. Correlations were calculated using Spearman's ρ. To analyze the contribution of the various factors predicting basal ganglia infarction a stepwise regression model was applied. Receiver operating characteristic (ROC) analysis was performed with DT as a test variable for the prediction of involvement of the lenticulostriate perforators as well as the involvement of the internal capsule. To determine the optimal threshold, the Youden Index was calculated. All data are shown as mean ± SD unless indicated otherwise. Statistical significance was assumed at *P* < 0.05.

## DISCLOSURES

None.
